# Genome Sequences of Four Strains of *Mycobacterium avium* subsp. *hominissuis,* Isolated from Swine and Humans, Differing in Virulence in a Murine Intranasal Infection Model

**DOI:** 10.1128/genomeA.00533-16

**Published:** 2016-06-16

**Authors:** N. Bruffaerts, C. Vluggen, L. Duytschaever, V. Mathys, C. Saegerman, O. Chapeira, K. Huygen

**Affiliations:** a Service “Immunology,” Operational Direction Communicable and Infectious Diseases, Scientific Institute of Public Health (WIV-ISP), Brussels, Belgium; b Service “Bacterial Diseases,” Operational Direction Communicable and Infectious Diseases, Scientific Institute of Public Health (WIV-ISP), Brussels, Belgium; c Unit “Bacterial Zoonoses of Livestock,” Operational Direction Bacterial Diseases, Veterinary and Agrochemical Research Centre (CODA-CERVA), Brussels, Belgium; d Research Unit in Epidemiology and Risk Analysis Applied to Veterinary Sciences (UREAR-ULg), Fundamental and Applied Research for Animal and Health (FARAH), Liège, Belgium; e Service Bioinformatique, Genoscreen, Lille, France

## Abstract

This paper announces the genome sequences of four strains of *Mycobacterium avium* subsp. *hominissuis*, isolated from cases of lymphadenopathy in swine and humans, differing in virulence in a murine intranasal infection model.

## GENOME ANNOUNCEMENT

Among nontuberculous mycobacteria (NTM), bacteria of the *Mycobacterium avium* complex are the most frequently isolated from patients (1, 2). The *M. avium* species is divided into four subspecies: *M. avium* subsp. *avium*, *M. avium* subsp. *silvaticum*, *M. avium* subsp. *paratuberculosis*, and *M. avium* subsp. *hominissuis* (3). These subspecies of *M. avium* are genetically very close, but they differ widely in their host range and pathogenicity. Indeed, *M. avium* subsp. *paratuberculosis* is responsible for an intestinal illness in ruminants known as Johne’s disease and may be a triggering factor of human Crohn’s disease. *M. avium* subsp. *avium* and *M. avium* subsp. *silvaticum* mainly infect birds, causing a tuberculosis-like disease, whereas *M. avium* subsp. *hominissuis* is a frequent agent of human and pig mycobacterioses (4), and an association between *M. avium* subsp. *hominissuis* and human lymphadenitis has been described (5). As *M. avium* subsp. *hominissuis* represents an increasing public health concern given its pathogenicity for both humans and pigs, detailed genotyping of human clinical isolates and swine isolates could contribute to establishing or excluding any epidemiological links between both hosts. Using variable-number tandem repeat analysis, it was recently reported that clinical *M. avium* subsp. *hominissuis* isolates exhibit geographical differences in genetic diversity, with isolates from Japan and Korea sharing a high degree of genetic relatedness, whereas isolates from the Netherlands and Germany were predominantly grouped in another cluster (2). Using multispacer sequence typing (MST) (6), we identified 46 different genotypes of *M. avium* subsp. *hominissuis* isolated among humans and pigs in Belgium, between 2011 and 2013 (7).

Using an intranasal infection model in BALB/c mice we compared the virulence of porcine and human isolates with different MST types (Bruffaerts et al, manuscript in preparation). Bacterial replication was monitored for 3 months by plating lung, spleen, and liver homogenates on Middlebrook 7H11 agar. Isolates varied significantly in virulence, with a human (12_062) and a porcine (LYM122) isolate of MST type 22 clearly showing higher bacterial numbers in lungs and more dissemination to spleen and liver than a human (12 _067) isolate and a porcine (LYM086) isolate of MST type 91.

Whole-genome sequencing was performed on these four isolates with an Illumina MiSeq (2 × 150-bp), and a quality analysis was realized using FastQC version 0.11.5. Assembly of the sequences in contigs was performed using Velvet version 1.2.1 and VelvetOptimiser.pl version 2.2.5. MyRast software was enabled to identify open reading frame regions, which were annotated using the database FigFams. Genome statistics are given in Table 1.

**TABLE 1:** Genome statistics of the four *M. avium* subsp. *hominissuis* isolates

Isolate	Accession no.	No. of *k*-mers	No. of contigs	Mean length (bp)	*N*_50_ (bp)	Total genome sequence length (bp)	No. of protein-coding sequences	G+C content (%)
12_062	FKJL01000001 to FKJL01000175	81	175	29,112	70,012	5,094,574	4,979	69
LYM122	FKJN01000001 to FKJN01000175	89	175	29,100	75,210	5,092,537	4,937	69
12_067	FKJO01000001 to FKJO01000216	91	216	23,649	63,538	5,108,242	4,910	69
LYM086	FKJM01000001 to FKJM01000199	81	199	26,672	72,334	5,307,771	5,131	69

### Nucleotide sequence accession numbers.

The four genome sequences have been deposited at the European Nucleotide Archive under the accession numbers listed in Table 1.
